# Evaluating the Efficacy of EcoSAFE: A Superoxidized Solution With HOCl at Neutral pH for Infection Control

**DOI:** 10.7759/cureus.102602

**Published:** 2026-01-29

**Authors:** Vasim Ahmad, Suresh Chopra, Anjali Goswami, Shashi Bala

**Affiliations:** 1 Microbiology, Rajan Babu Institute of Pulmonary Medicine and Tuberculosis, New Delhi, IND; 2 Pulmonology, Rajan Babu Institute of Pulmonary Medicine and Tuberculosis, New Delhi, IND

**Keywords:** ecosafe, hospital-acquired infections, hypochlorous acid, infection control, microbial contamination, neutral ph disinfectant, superoxidized solution, surface disinfection

## Abstract

Background

Hospital-acquired infections (HAIs) pose a major challenge to healthcare systems, increasing patient morbidity, mortality, and costs. Contaminated air and surfaces in hospitals serve as key sources of infection transmission. Developing safe, reliable, and efficient disinfection methods is essential to minimizing microbial contamination in these environments.

Objective

This study assessed the effectiveness of EcoSAFE superoxidized solution containing hypochlorous acid (HOCl) at neutral pH in reducing microbial contamination and improving infection control in hospital settings.

This study aimed to assess the effectiveness of EcoSAFE in preventing and controlling infections in high and moderate-risk areas within hospital settings. The results highlight EcoSAFE's serve as a safer, practical and more efficient alternative to conventional disinfectants for infection control. Therefore, EcoSAFE will be of great interest in the development of continuous room disinfection methods, surface disinfectants and equipment cleaning in hospitals.

Methods

The evaluation was carried out at the Rajan Babu Institute of Pulmonary Medicine and Tuberculosis Hospital (RBIPMTH), New Delhi. EcoSAFE was applied for routine disinfection of operation theaters and hospital surfaces by both spraying and fumigation. Air and surface samples were collected before and after treatment, and microbial counts were determined by measuring colony-forming units (CFUs) to assess the reduction in bacterial and fungal load. A total of 1,056 surface swab samples (528 pre- and 528 post-disinfection) and 960 air-exposed plates were analyzed. Microbial load was assessed before and after treatment by quantifying colony-forming units (CFUs).

Results

Following the EcoSAFE application, there was a 100% reduction in growth on all 528 post‑fumigation surface swabs and up to 100% reduction in airborne colony counts in both high‑ and moderate‑risk areas. The solution demonstrated rapid antimicrobial action, achieving significant reduction within minutes of contact. Disinfection did not require the closure of operation theaters, and no odor, irritation, or post-cleaning residues were noted. The product was convenient to handle, non-corrosive, and entirely safe near hospital personnel and patients; no personal protective equipment (PPE) was needed during use.

Conclusion

EcoSAFE proved to be an effective, safe, and easy-to-use disinfectant for healthcare environments. Its quick action, neutral pH, and absence of chemical residues make it a practical option for routine use. Adoption of EcoSAFE in hospital infection control programs may help reduce microbial contamination and contribute to the prevention of HAIs.

## Introduction

Hospital-acquired infections (HAIs), also known as nosocomial infections, are defined as infections that arise 48 hours or more after admission, or become apparent within three days after discharge, or within 30 days of a surgical procedure [1]. These infections are not present or incubating at the time of admission, confirming that they originate from exposure within healthcare facilities [1,2]. HAIs continue to be a major concern for patient safety. They contribute to extended hospital stays, increased treatment costs, and higher rates of illness and mortality. Their global burden is significant. Approximately one in ten patients worldwide develops an HAI during hospitalization, and the impact is markedly higher in low- and middle-income countries [3]. In India, estimated HAI rates range from 10% to 20% among admitted patients, highlighting the scale of the problem [4]. At the global level, the prevalence of HAIs has been reported as 0.14%, with an annual rise of 0.06%, indicating a gradual upward trend [5]. This burden is particularly difficult to manage in developing countries, where healthcare systems often face resource limitations. Notable disparities exist between regions. High-income countries report HAI rates of 4.5 to 7.1 per 100 patients. In contrast, low- and middle-income countries experience substantially higher rates, averaging 15.5 per 100 patients. Intensive care units (ICUs) show the greatest imbalance. ICUs in low- and middle-income countries (LMICs) experience three times more HAIs than those in developed nations [6,7]. These patterns underscore the persistent challenge posed by HAIs and the critical need for effective infection-prevention measures, especially in resource-limited healthcare systems.

Inadequate hygiene standards, together with the increasing burden of hospitalized patients, play a major role in the spread of these infections [8]. The hospital environment poses a high risk for microbial contamination. This leads to HAIs, prolonged hospital stays, and increased healthcare costs [9]. Most HAIs are transmitted through contaminated surfaces, medical equipment, or the hands of healthcare workers. Therefore, proper disinfection practices are essential in minimizing infection spread [10].

Currently, hospitals use a range of disinfectants, including alcohol-based solutions, hydrogen peroxide, quaternary ammonium compounds (QACs), and chlorine-based products. These agents are effective, but each has important limitations. Alcohol-based disinfectants evaporate rapidly and do not provide long-term surface protection [11]. Hydrogen peroxide is potent, yet it often requires high concentrations that may cause irritation and surface corrosion [12]. QACs are widely used; however, they show limited activity against spores and certain viruses [13]. Chlorine-based disinfectants, such as sodium hypochlorite, can produce harmful by-products and degrade over time, reducing their effectiveness [14].

Moreover, there is a lack of sufficient hospital-based evidence evaluating safer disinfectant alternatives, particularly superoxidized water (SOW) formulations-under real-world conditions across different risk-graded clinical areas. Unlike several hypochlorous acid-based solutions that show rapid loss of activity, EcoSAFE exhibits improved stability, supporting sustained antimicrobial performance. This gap underscores the need for systematic assessment of such agents in operational hospital environments.

EcoSAFE, a superoxidized water (SOW), provides broad-spectrum antimicrobial action while being non-toxic, non-corrosive, and environmentally friendly [15,16]. It starts working within 30 seconds against bacteria, viruses, fungi, and spores without causing harm to humans or surfaces. Furthermore, its stability over time ensures prolonged efficacy, unlike other HOCl solutions that degrade quickly [17].

This study aimed to assess the effectiveness of EcoSAFE in preventing and controlling infections in high and moderate risk area within hospital settings. The results highlight EcoSAFE's serve as a safer, practical and more efficient alternative to conventional disinfectants for infection control. Therefore, EcoSAFE will be of great interest in the development of continuous room disinfection methods, surface disinfectants and equipment cleaning in hospitals.

## Materials and methods

Study site and duration

This was a prospective interventional observational study conducted in the Department of Microbiology, RBIPMTH, New Delhi, India. Fumigation was performed weekly (every Saturday), and environmental samples were collected 30 minutes after fumigation on a weekly basis over six months from October 2024 to March 2025. Sampling occurred under sterile conditions in hospital sections categorized by risk: high-risk (minor operating theater and bronchoscopy theater) and moderate-risk (microbiology laboratory and sample collection room). Since this study involved only environmental sampling (air and surfaces) without the use of human participants or identifiable data, it was exempted from IRB/Ethics Committee approval. 

EcoSAFE is a ready-to-use disinfectant containing the active ingredient Hypochlorous acid (HOCl) at a concentration of 0.004%, with super-oxidized water constituting 99.97% of the formulation. No dilution was required. All designated areas were first thoroughly cleaned with soap and water, after which EcoSAFE disinfectant was used for surface disinfectant and fumigation. For surface disinfection, the spray bottle was positioned 4-6 inches from the target, delivering even coverage through sweeping motions to ensure complete wetting, with a 5-10-minute contact time before air-drying or wiping with a sterile cloth. 

For fumigation, EcoSAFE was applied using a fogger machine for a standardized exposure period as per room size, 4 liters in 10 minutes for 180 cubic meters. Keep the fogger machine 1.5-2 meters away from the surfaces. Move the machine slowly, ensuring uniform distribution of the disinfectant across surfaces, walls, ceilings, and the surrounding airspace. 

Sampling

In selected areas, air and surface samples were collected before fumigation and immediately after 30 minutes of EcoSAFE fumigation to assess its efficacy. 

Surface Sampling

Surface sampling was done by taking a swab from the OT surfaces. Two types of surfaces were chosen in our study for sampling, like: In rough surfaces (include walls), stainless steel surfaces (include tables, trolleys, Crush Cart, OT Bed, OT Table).

Air Sampling

Air samples were taken by the settle plate method by placing the Plates on four corners and the centre for 30 min. Samples were appropriately labelled and immediately transferred and processed in the microbiology laboratory.

Data collection

The samples were collected for a period of 6 months from different high, moderate risk areas of the hospital before and after 30 minutes of fumigation with the use of EcoSAFE disinfectant. The efficacy of the disinfectant can be calculated on the basis of log reduction by comparing the results of pre- and post-fumigation. Log reduction was calculated by using the following formula:

RF = Log_10 _- (Prevalue) - Log_10_ (Postvalue)

Culture

Swabs taken from different areas were cultured for bacteriological growth on blood agar (BA) plates and incubated up to 48 hours at 37^0^C while fungal culture, the Sabouraud Dextrose Agar (SDA) plates, were incubated for 14 days at 27^0^C The colonies were enumerated and translated into colony-forming units per cubic meter (CFU/m^3^) by using Omeliansky formula [18]. Environmental monitoring was done by exposing blood agar plates for 30 minutes at four corners and the center of the room, followed by incubation for 24 hours at 37°C.

Microscopy

The microscopy method was used to identify bacterial & fungal colonies. Gram’s Staining was performed under oil immersion using an Olympus CX21i microscope for bacterial smear, and lacto-phenol cotton blue staining was done for fungal smear. Along with microscopy, various biochemical tests were used to identify the isolates, like catalase, coagulase, oxidase, etc.

Statistical analysis

Data were summarized as means with standard deviations; where applicable, pre‑ and post‑fumigation CFU counts were compared using appropriate non‑parametric/parametric tests, and p‑values with confidence intervals are now reported.

## Results

Surface samples results

During the study, 1,056 swab samples were collected from high-and moderate-risk areas (528 before and 528 after fumigation). Out of 528 pre-fumigation swab samples, 288 were from high-risk areas,167 (57.9%) samples were culture positive, and 121(42%) were negative for bacterial growth on Blood Agar (Table 1). While 2 (0.6%) samples were found to be fungal positive, and 266 (92.4%) samples were sterile on SDA media for fungal growth before fumigation. Pre-fumigation colony counts totaled 393 across 167 positive swab samples (Table 1). Among these, 40% were Micrococcus spp., 22.6% Gram-positive coccobacilli, while common pathogens seen were Pseudomonas spp. 15% followed by Staphylococcus aureus 11%, Klebsiella pneumoniae 9%, and Candida sp. 2.4%. All 528 post-fumigation swabs were negative for bacterial and fungal growth. 

**Table 1:** Shows the swab data of bacterial and fungal load of high-risk area (OTs) before and after fumigation. BA: Blood Agar; SDA: Sabouraud Dextrose Agar; OT: Operation theatre BA: * P<0.000001, P=1 95% CI (0.86-1.00) SDA: p-value and CI NA

Site	BA (Pre-fumigation)			BA (Post-fumigation)		
	N=288			N=288		
	No Growth	Growth	Total no. of colony	No Growth	Growth	Total no. of Colony
Crush cart (N=48)	16	32	86	48	0	0
OT Bed (N=48)	19	29	69	48	0	0
OT Table (N=48)	23	25	48	48	0	0
Trolly (N=48)	17	31	60	48	0	0
Wall-1 (N=48)	24	24	71	48	0	0
Wall-2 (N=48)	22	26	59	48	0	0
Sites	SDA (Pre-fumigation)			SDA (Post-fumigation)		
	N=288			N=288		
	No Growth	Growth	Total no. of colony	No Growth	Growth	Total no. of colony
Crush cart (N=48)	48	0	0	48	0	0
OT Bed (N=48)	48	0	0	48	0	0
OT Table (N=48)	48	0	0	48	0	0
Trolly (N=48)	48	0	0	48	0	0
Wall-1 (N=48)	47	1	2	48	0	0
Wall-2 (N=48)	47	1	8	48	0	0

While in moderate-risk areas, before fumigation total of 240 swab samples were collected. Among them, 101(42%) swab samples were positive for bacterial growth on Blood agar, while 139(57.9%) samples were negative for bacterial culture (Table 2). None of the culture was positive after fumigation. In the moderate-risk area, no fungal growth was seen in any pre- and post-culture plates (Table 2). In moderate-risk areas most prevalent were nonpathogenic microorganisms, i.e, Micrococcus species 60% followed by 35% Gram-positive coccobacilli and 5% Pseudomonas sp. (Pathogenic). None of the swab samples showed growth after fumigation.

**Table 2:** Shows the swab data of bacterial and fungal load of moderate-risk area (laboratory and sample collection) before and after fumigation. BA: Blood Agar; SDA: Sabouraud Dextrose Agar BA: * P<0.000001, P=1 95% CI (0.86-1.00) SDA: P value and CI NA

Sites	BA (Pre-fumigation)			BA (Post-fumigation)		
	N=240			N=240		
	No Growth	Growth	Total no. of Colony	No Growth	Growth	Total no. of Colony
Table-1 (N=48)	24	24	29	48	0	0
Table-2 (N=48)	24	24	32	48	0	0
Table-3 (N=48)	26	22	29	48	0	0
Table-4 (N=48)	35	13	17	48	0	0
Trolly (N=24)	16	8	11	24	0	0
Slab(N=24)	14	10	14	24	0	0
Site	SDA (Pre-fumigation)			SDA (Post-fumigation)		
	N=240			N=240		
	No Growth	Growth	Total no. of Colony	No Growth	Growth	Total no. of Colony
Table-1 (N=48)	48	0	0	0	0	0
Table-2 (N=48)	48	0	0	0	0	0
Table-3 (N=48)	48	0	0	0	0	0
Table-4 (N=48)	48	0	0	0	0	0
Trolly (N=24)	24	0	0	0	0	0
Slab (N=24)	24	0	0	0	0	0

Air sampling results

A total of 960 plates were exposed during the study. The percentage reduction of microorganisms was shown to be up to 100% in different high-risk and moderate-risk areas. Percentage reduction and average no. of colonies pre and post fumigation in high-risk areas on BA and SDA for the growth of bacteria and fungi (Table 3). In high-risk areas, 80% were non-pathogenic Micrococcus spp. and 17% were Gram-positive coccobacilli. At the same time, only 3% were Pathogenic Pseudomonas spp. After fumigation, no pathogenic microorganism was shown to grow.

**Table 3:** Air sampling results of high-risk areas (broncho & minor) OT pre and post fumigation with percentage of reduction. BA: Blood Agar; SDA: Sabouraud Dextrose Agar

Month	BA (Broncho OT)		Percentage of Reduction	Log Reduction	BA (Minor OT)		Percentage of Reduction	Log Reduction
	Average number of colony Pre-Fumigation	Average number of colony Post-Fumigation			Average number of colony Pre-Fumigation	Average number of colony Post-Fumigation		
October	24.9	0.53	98%	1.70	8.4	0.26	97%	1.5
November	16.2	0	100%	≥1.2	0.7	0	100%	≥0
December	3.5	0	100%	≥0.5	1.55	0	100%	≥0.2
January	7.9	0	100%	≥0.9	3.25	0	100%	≥0.5
February	1.6	0	100%	≥0.2	1.45	0	100%	≥0.2
March	2.32	0	100%	≥0.4	1.04	0	100%	≥0
Month	SDA (Broncho OT)		Percentage of Reduction	Log Reduction	SDA (Minor OT)		Percentage of Reduction	Log Reduction
	Average number of colony Pre-Fumigation	Average number of colony Post-Fumigation			Average number of colony Pre-Fumigation	Average number of colony Post-Fumigation		
October	0.4	0	100%	NA	0	0	100%	NA
November	0.1	0	100%	NA	0	0	100%	NA
December	0.1	0	100%	NA	0.1	0	100%	NA
January	0.2	0	100%	NA	0.05	0	100%	NA
February	0	0	100%	NA	0.05	0	100%	NA
March	0.1	0	100%	NA	0.08	0	100%	NA

In the moderate-risk area, percentage reduction and average no. of colonies of bacteria and fungus pre and post fumigation were shown in (Table 4). In the moderate-risk area, 23% were found to be coccobacilli, 72% were Micrococcus, and 5% were Pseudomonas spp. post-fumigation, no growth was seen in the moderate-risk area.

**Table 4:** Air sampling results of moderate-risk areas (microbiology lab & sample room) pre and post fumigation with percentage of reduction. BA: Blood Agar; SDA: Sabouraud Dextrose Agar

Month	BA (Broncho OT)		Percentage of Reduction	Log Reduction	BA (Minor OT)		Percentage of Reduction	Log Reduction
	Average number of colony Pre-Fumigation	Average number of colony Post-Fumigation			Average number of colony Pre-Fumigation	Average number of colony Post-Fumigation		
October	15.8	0	100%	≥1.2	7.8	0	100%	≥0.9
November	12.67	0	100%	≥1.1	1.25	0	100%	≥0.1
December	4.1	0	100%	≥0.6	1.4	0	100%	≥0.2
January	2.5	0	100%	≥0.4	1.8	0	100%	≥0.3
February	0.72	0	100%	NA	3.4	0	100%	≥0.5
March	0.65	0	100%	NA	0.6	0	100%	NA
Month	SDA (Broncho OT)		Percentage of Reduction	Log Reduction	SDA (Minor OT)		Percentage of Reduction	Log Reduction
	Average number of colony Pre-Fumigation	Average number of colony Post-Fumigation			Average number of colony Pre-Fumigation	Average number of colony Post-Fumigation		
October	0.75	0	100%	NA	0.2	0	100%	NA
November	0.86	0	100%	NA	0	0	100%	NA
December	0	0	100%	NA	0	0	100%	NA
January	0	0	100%	NA	0	0	100%	NA
February	0	0	100%	NA	0	0	100%	NA
March	0	0	100%	NA	0	0	100%	NA

## Discussion

The results of this study provide strong evidence supporting the efficacy of EcoSAFE, a superoxidized solution containing neutral pH hypochlorous acid (HOCl), as a powerful disinfectant in hospital settings. The complete elimination of microbial growth in both air and surface samples after EcoSAFE application across high & moderate-risk areas demonstrates its potential to outperform traditional disinfectants in practical healthcare environments.

Its broad-spectrum antimicrobial activity is central to its effectiveness. The complete elimination of Pseudomonas spp., Acinetobacter, Staphylococcus aureus (MSSA and MRSA), Candida spp., and Micrococcus spp. indicates that EcoSAFE acts on both pathogenic and non-pathogenic organisms, including resistant hospital strains. This supports previous literature suggesting that HOCl-based disinfectants exhibit rapid and potent activity against a wide range of pathogens, including bacteria, fungi, viruses, and even spores [15,17,19].

Compared to conventional disinfectants like alcohol-based solutions, quaternary ammonium compounds (QACs), hydrogen peroxide, and sodium hypochlorite, EcoSAFE provides several operational and safety advantages. For example, alcohol evaporates quickly and lacks residual activity, limiting its utility in long-term contamination control [11]. QACs, while widely used, have been shown to be less effective against spores and enveloped viruses, and frequent use may contribute to microbial resistance [10]. Hydrogen peroxide, though effective, can be irritating to skin and mucous membranes and may damage delicate medical equipment [12]. Sodium hypochlorite, though effective, poses significant drawbacks such as corrosiveness, toxic by-products, and instability over time [14].

In contrast, EcoSAFE’s non-corrosive, odorless, and non-irritating nature makes it safer for use around patients and staff [20,21,22]. Its neutral pH formulation is particularly advantageous, as acidic HOCl solutions are often unstable and degrade rapidly upon exposure to air or light. This stability allows for consistent disinfection efficacy over time, as observed in this study over a six-month duration. Similar findings on HOCl stability and safety were reported by [15,17], who highlighted HOCl’s environmental compatibility and absence of toxic residues.

A unique advantage noted in this study was the elimination of downtime. Unlike traditional fumigants, which may require area closure for hours due to toxic residues or strong odors, EcoSAFE allowed rapid re-entry into disinfected areas, facilitating uninterrupted clinical workflow. This is critical in high-traffic areas such as operation theatres and ICUs, where time-sensitive care must not be delayed.

The air sampling data, showing 94% to 100% reduction in colony-forming units (CFUs), further confirms EcoSAFE’s effectiveness in reducing airborne pathogens. These results are particularly relevant in the context of HAIs, as airborne transmission-especially in closed settings like bronchoscopy suites-can lead to nosocomial outbreaks [10]. The high reduction in fungal CFUs also suggests that EcoSAFE may help reduce the risk of opportunistic fungal infections in immunocompromised patients.

From a practical perspective, the ease of application (via fogging) and the no need for secondary cleaning add to EcoSAFE’s usability in real-world hospital settings. Surfaces were cleaned once and fogged with EcoSAFE, achieving disinfection without additional labor or chemical layering. This is consistent with [9], who emphasize that simplicity and consistency in cleaning protocols significantly impact HAI prevention.

We also saw better staff adherence to infection control, along with steady improvements in daily cleaning. This included using EcoSAFE on high-touch surfaces and weekly fumigation with EcoSAFE. Together, these changes lowered the microbial load over time.

Despite the promising results, this study's findings must be interpreted within the scope of its methodology. While the results clearly show EcoSAFE’s efficacy over six months, longer-term studies could help assess the sustainability of microbial control and the possibility of microbial resistance.

This study has limitations as it was conducted at one hospital without comparisons to other disinfectants. The findings are based only on air and surface samples, so the impact on actual hospital infection rates could not be assessed. We also did not use advanced molecular tests, and viruses or spore-forming microbes were not specifically checked, which may limit the overall scope of the results.

## Conclusions

The study confirms that EcoSAFE is a highly effective and safe disinfectant for hospital infection control. Its rapid pathogen elimination, combined with its eco-friendly profile, positions it as a promising alternative to conventional disinfectants. An added operational advantage is the absence of downtime, enabling uninterrupted clinical workflows in critical areas such as operating theatres and bronchoscopy suites. By efficiently reducing microbial load, EcoSAFE also holds significant potential in preventing healthcare-associated infections (HAIs), CLABSI infection, particularly in high-risk and immunocompromised patient care settings. Collectively, these attributes make EcoSAFE a strong candidate for adoption as a standard disinfection solution in modern healthcare facilities. To further validate its superiority over existing market disinfectants, a multicenter comparative study is recommended.
